# Metabolic power in hurling with respect to position and halves of match-play

**DOI:** 10.1371/journal.pone.0225947

**Published:** 2019-12-31

**Authors:** Damien Young, Shane Malone, Kieran Collins, Laurent Mourot, Marco Beato, Giuseppe Coratella

**Affiliations:** 1 Limerick Institute of Technology, Thurles Campus, Thurles, Tipperary, Ireland; 2 Gaelic Sports Research Centre, Technological University Dublin, Tallaght, Dublin, Ireland; 3 The Tom Reilly Building, Research Institute for sport and exercise sciences, Liverpool John Moores University, Liverpool, United Kingdom; 4 Research Unit EA3920 Prognostic Markers and Regulatory Factors of Cardiovascular Diseases and Exercise Performance, Exercise Performance Health, Innovation Platform, University of Bourgogne Franche-Comté, Besancon, France; 5 Tomsk Polytechnic University, Tomsk, Russia; 6 School of Health and Sports Science, University of Suffolk, Ipswich, United Kingdom; 7 Department of Biomedical Sciences for Health, University of Milan, Italy; Universidade Federal de Juiz de Fora, BRAZIL

## Abstract

The current investigation compared the metabolic power and energetic characteristics in team sports with respect to positional lines and halves of match-play. Global positioning system (GPS) technology data were collected from 22 elite competitive hurling matches over a 3-season period. A total of 250 complete match-files were recorded with players split into positional groups of full-back; half-back; midfield; half-forward; full-forward. Raw GPS data were exported into a customized spreadsheet that provided estimations of metabolic power and speed variables across match-play events (average metabolic power [P_met_], high metabolic load distance [HMLD], total distance, relative distance, high-speed distance, maximal speed, accelerations, and deceleration). P_met_, HMLD, total, relative and high-speed distance were 8.9 ± 1.6 W·kg^-1^, 1457 ± 349 m, 7506 ± 1364 m, 107 ± 20 m·min^-1^ and 1169 ± 260 m respectively. Half-backs, midfielders and half-forwards outperformed full-backs (Effect Size [ES] = 1.03, 1.22 and 2.07 respectively), and full-forwards in P_met_ (Effect Size [ES] = 1.70, 2.07 and 1.28 respectively), and HMLD (full-backs: ES = -1.23, -1.37 and -0.84 respectively, and full-forwards: ES = -1.77, -2.00 and -1.38 respectively). Half-backs (ES = -0.60), midfielders (ES = -0.81), and half-forwards (ES = -0.74) experienced a second-half temporal decrement in HMLD. The current investigation demonstrates that metabolic power may increase our understanding of the match-play demands placed on elite hurling players. Coaches may utilize these findings to construct training drills that replicate match-play demands.

## Introduction

Research in team sport has provided valuable information about the movement demands of elite players during match-play describing the different locomotion intensities ranging from low to high speeds [1–3]. The emergence of player tracking technology has facilitated the capability to assess the match-play locomotion ranging from walking to sprinting as reported in soccer, rugby and Australian football [2,4–6]. Consequently, global positioning system (GPS) technology has been used to quantify the positional profile and temporal changes during match-play [2,4–6]. These studies have focused on presenting distances covered using fixed absolute speed-based thresholds (e.g. high-speed running: ≥ 17 km·h^-1^) allowing for an estimation of the match-play demands [1,7–9]. Given the start-stop nature of team sports, players’ changes in speed may not be accounted for within these fixed high-speed thresholds. Therefore, quantifying the number of accelerations and decelerations has gained interest in team sports, as they help to determine the number of transitions between the speed thresholds and even the changes in speed within the sprint threshold [7,10,11]. Indeed, accelerating, even at low-speed, is demanding per se [12].

While it is difficult to measure directly the exact energy cost of changing speed, a metabolic power calculation based on a theoretical model has been used to estimate the energy cost of acceleration and deceleration in team sports [7,8,10,12,13]. This model proposes that accelerated running on level ground is energetically equivalent with that of running uphill at constant speed [14]. Therefore, once speed and acceleration are known, the metabolic power output can be calculated [12]. Accordingly, metabolic power analyses have been conducted in soccer [12], field hockey [10], Australian football [7], Rugby League [13] and Gaelic football [8]. These investigations provided additional insight to previous studies which have employed GPS time-motion analyses of activity demands of training and match-play [1,4].

Despite the aforementioned studies that used metabolic power estimates, some concerns have been raised about its validity and reliability to provide energy cost estimates similar to those obtained through analysis performed by the gas analyser [15–17]. However, a previous study in soccer has provided evidence for concurrent ecological validity to this approach, reporting very large correlations between aerobic fitness variables and metabolic power estimates of high-power distance during professional matches [18]. Moreover, other studies have shown that metabolic power estimates can be sensitive to decrements in running performance during competition measured by using GPS [8,10,19]. Additionally, these metabolic power estimates were shown to account for positional differences and temporal decrement changes in match running performance [8,19]. Therefore, the combination of the metabolic power approach and GPS time-motion analysis should be used to present a description of the intermittent running demands that include accelerations and decelerations [16].

Currently, the metabolic match-play profile of soccer [12], field hockey [10], Australian football [7], Rugby League [13] and Gaelic football [8] have been presented. However, a similar team sport called hurling has yet to be investigated. Hurling is a physically demanding and highly skilled stick and ball field sport, consisting of changes of direction, tackling, jumping and sprint actions [1,6,20]. The game is 70 minutes (35 minutes per half) in duration and is played on a pitch 140 m long and 90 m wide [1]. Two teams of 15 players (1 goalkeeper and 14 outfield players) contest for possession; through high-intensity action players aim to create space for team-mates in order to facilitate scoring chances to influence the score-line in their favor [21]. Players’ physical, tactical, and technical roles differ between the 5 distinctive positions (full backs, half backs, midfielders, half forwards, and full forwards) [21]. Players, each representing a county, compete for Provincial and All-Ireland senior championship, which attracts large attendances of over 80,000 spectators for the final [1].

Similar to other team sports, the match-play demands of hurling have been investigated using GPS [1–3,5,6]. The combination of metabolic power metrics with GPS metrics would help to provide a more complete profile of the match demands of hurling. Specifically, knowledge of the high-powered activities such as accelerations and decelerations not recorded by traditional GPS speed zones would help coaches to design sport-specific conditioning games (e.g. small-sided games) [22]. Furthermore, power and high-intensity activities have been previously shown to provide intense training stimuli in professional team sport athletes providing both physiological and neuromuscular adaptations [23]. In addition, previous research strongly support that these activities that include changes in speed could be implemented throughout training sessions to obtain sport specific metabolic adaptations so that players are able to minimize the fatigue related decrements in performance during official games [1,24]. As hurling match-play is shown to be demanding [1], knowledge of the metabolic power profile would provide further information about the nutritional strategies required both pre-match and at half-time so that players are fueled to perform for the full duration of match-play [8,25]. However, no investigation has described the metabolic power demands of elite hurling match-play associated with GPS time motion analysis. Therefore, the aims of the current study were to, 1) describe the metabolic variables of elite hurling match-play with respect of positional groups and 2) to examine the temporal profiles of these measures across halves of match-play. It was hypothesized that there would be differences in metabolic power variables between positions and between playing halves.

## Materials and methods

### Participants

Thirty-six (*n = 36*) elite male hurlers (mean ± SD, age: 27 ± 4 years, height: 181 ± 5 cm, mass: 86 ± 4 kg) volunteered to partake in the current observational investigation. To be considered as elite, each player has been selected from this club to join the county team as previously described [1,6]. Specifically, they competed at the highest level (Provincial and All-Ireland Championship) according to the Gaelic Athletic Association rules [1,5,6]. The players were classified according to their playing position during each match resulting in the following number of data sets per position: full backs: *n = 50*, half backs: *n = 50*, midfielders: *n = 50*, half forwards: *n = 50*, and full forwards: *n = 50*. Only those who were free from injury and illness were eligible to partake in the study. The players were informed of the purpose, benefits, and potential risks of the study. Written informed consent and medical declaration were obtained from all participants. Finally, the University Bourgogne Franche-Comté Ethics Committee approved all procedures, and the study was conducted according to the Declaration of Helsinki (1975) for studies involving human subjects.

### Procedures

**Fig 1:**
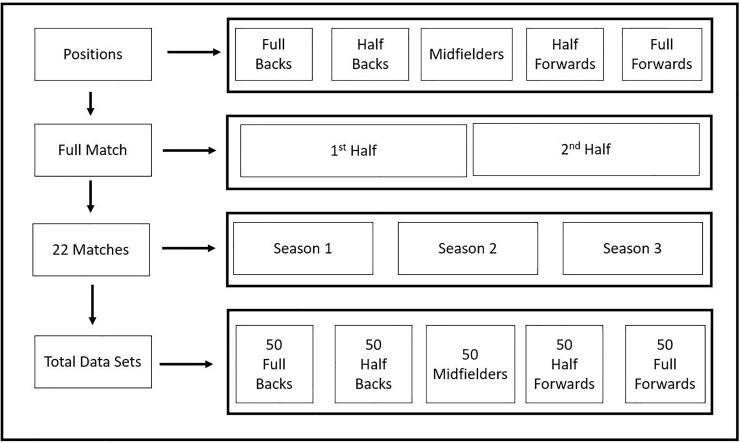
Details of the experimental design. The participants were divided into five different playing positions. Data were collected over the 3 seasons resulting in 250 individual data sets.

The current study was designed to examine the metabolic power variables of elite hurling players with respect to position and halves during competition. The sample size was based on previous hurling studies [1,6]. Data were collected during 22 games across 3 full competition seasons (February 2016 –August 2018) resulting in 250 individual samples being collected (Fig 1). Data were included only if a full match (70 minutes) was completed. GPS was used to quantify players’ running performance during competitive games. All competitive matches took place between 14.00 and 18.00 hours. The players were requested to abstain from strenuous physical activity in the 24 hours before competitive matches and to report to the game fully hydrated [1].

The players’ movements were measured using GPS sampling at 10-Hz (STATSports Viper Pod, Newry, Northern Ireland). The GPS device was encased within a protective harness between the player’s shoulder blades in the upper thoracic-spine region [1,26]. Before entering the field of play GPS devices were fixed to the athletes, the device was then activated and satellite lock established for a minimum of 15 minutes before the commencement of each match [27]. All players wore the same GPS unit for each match during the seasons analysed to minimize inter-unit error [28–30]. The validity and reliability of this device have previously been reported [30,31]. After the completion of each match, GPS data were downloaded to a computer through the bespoke STATSport analysis software (STATSport Viper Firmware 2.28) to be stored and analysed. Each file was trimmed so that data recorded only when the player was on the field were included for further analysis. The proprietary software provided instantaneous raw velocity data at 0.10-second intervals, which was then exported and placed into a customized Microsoft Excel spreadsheet (Microsoft, Redmond, WA, USA). This customized spreadsheet allowed for the calculation of traditional speed-based measures such as: total distance (m), relative distance (m·min^-1^), high-speed distance (m: ≥ 17 km·h^-1^), sprint distance (m: ≥ 22 km·h^-1^), N° accelerations (> 2 m^.^s^-2^), N° decelerations (< 2 m^.^s^-2^) [32], and maximal velocity (km·h^-1^) [4] (e.g. S1 Table). Furthermore, the spreadsheet allowed for estimation of average metabolic power (P_met_: W·kg^-1^) and power across 6 zones: minimal power (> 0–5 W·kg^-1^), low power (> 5–10 W·kg^-1^), intermediate power (> 10–15 W·kg^-1^), moderate power (> 15–25 W·kg^-1^), high power (> 25 to 50 W·kg^-1^), elevated power (> 50 W·kg^-1^), and HMLD (> 25 W·kg^-1^) [8].

The indirect estimation of the P_met_ used the rationale that accelerated running on flat terrain is energetically analogous to uphill running at a constant speed [12]:

$$EC\;\left(J\cdot kg^{‐1}\cdot m^{‐1}\right)=\left(155.4\;ES^{5}‐30.4\;ES^{4}‐43.3\;ES^{3}+46.3\;ES^{2}+19.5\;ES+3.6\right)EM$$

Where EC is the energy cost of accelerated running on grass, EM is the equivalent mass and ES is the equivalent slope. For further clarification about the rationale of this algorithm, please see Osgnach et al. [12].

### Statistical analysis

Data are presented as mean ± SD and 95% confidence intervals (CI). Descriptive analysis and assumptions of normality were verified before parametric statistical analysis. The dependent variables across the range of analysis were, P_met_, power across 6 zone (minimal power, low power, intermediate power, moderate power, high power, elevated power), HMLD, total distance (m), relative distance (m·min^-1^), high-speed distance (m), sprint distance (m), accelerations (n), decelerations (n), and maximal velocity with match periods and playing positions as independent factors. The analysis was performed using a two-way (position x half) mixed design (ANOVA). When significant F-values were found, post hoc analysis was performed (with Bonferroni corrections applied to the alpha value). Statistical significance was set at *a* ≤ 0.05. Cohen’s effect size (*d*) was used to describe the differences in running performance across positions and halves of play and was categorized with d < 0.20, 0.20–0.59, 0.60–1.19, 1.20–1.99, and ≥ 2.00 and interpreted as follows: *trivial*, *small*, *moderate*, *large*, and *very large* differences, respectively [33]. Statistical analysis was performed using SPSS version 22.0 (IBM Corp, Armonk, NY, USA).

## Results

The descriptive statistics for the metabolic power variables (P_met_, power zones, and HMLD) and distance variables (total distance, relative distance, high-speed distance, sprint distance, maximal speed, accelerations and decelerations) are presented in Table 1.

**Table 1:** The metabolic power and distance variables during elite hurling match-play with respect of the first and second halves of play. Data are presented as mean ± SD, Difference (95% CI) and Effect size.

	Full Game	1^st^ Half	2^nd^ Half	Difference 95% CI	Effect Size
***Metabolic Power Variables***					
Average Metabolic Power (P_met_: W·kg^-1^)	8.9 ± 1.6	9.4 ± 2.2	8.1 ± 2.5 *	-1.3 (-1.7 to -1.0)	0.55 (S*mall*)
MP Distance (m: > 0–5 W·kg^-1^)	1092 ± 217	553 ± 103	538 ± 156	-14 (-36 to -7)	0.11 (*Trivial*)
LP Distance (m: > 5–10 W·kg^-1^)	2340 ± 431	1208 ± 223	1131 ± 336 *	-78 (-131 to -26)	0.27 (S*mall*)
IP Distance (m: > 10–15 W·kg^-1^)	1076 ± 275	576 ± 154	501 ± 176 *	-77 (-102 to -51)	0.45 (S*mall*)
MDP Distance (m: > 15–25 W·kg^-1^)	1517 ± 522	805 ± 289	713 ± 310 *	-97 (-137 to -55)	0.31 (S*mall*)
HP Distance (m: > 25 to 50 W·kg^-1^)	1073 ± 320	569 ± 180	504 ± 199 *	-69 (-97 to -41)	0.34 (S*mall*)
EP Distance (m: > 50 W·kg^-1^)	385 ± 96	208 ± 56	178 ± 63 *	-31 (-41 to -21)	0.50 (S*mall*)
HMLD (m: > 25 W·kg^-1^)	1457 ± 349	776 ± 193	681 ± 232 *	-96 (-134 to -66)	0.45 (S*mall*)
***Distance Variables***					
Total Distance (m)	7506 ± 1364	3930 ± 666	3576 ± 1018 *	-336 (-514 to -219)	0.41 (Small)
Relative Distance (m·min^-1^)	107 ± 20	112 ± 20	102 ± 29 *	-10 (-14 to -6)	0.40 (S*mall*)
High-Speed Distance (m: ≥ 17 km^.^h^-1^)	1169 ± 260	612 ± 162	557 ± 171 *	-59 (-90 to -27)	0.33 (*Small*)
Sprint Distance (m: ≥ 22 km^.^h^-1^)	350 ± 93	188 ± 74	162 ± 65 *	-27 (-43 to -11)	0.37 (S*mall*)
Maximal Speed (km^.^h^-1^)	29.1 ± 2.1	29.3 ± 2.3	29.0 ± 3.0	-0.3 (-0.8 to 0.1)	0.11 (*Trivial*)
Accelerations (> 2 m.s^-2^) (n)	126 ± 25	66 ± 13	61 ± 18 *	-5 (-9 to -2)	0.32 (S*mall*)
Decelerations (< 2 m.s^-2^) (n)	120 ± 26	63 ± 14	58 ± 18 *	-5 (-8 to -2)	0.31 (S*mall*)

MP = Minimal Power; LP = Low Power; IP = Intermediate Power; MDP; Moderate Power; HP = High Power; HMLD = High Metabolic Load Distance; Diff = Difference, CI = Confidence interval, ES = Effect size.

* Significantly different (p < 0.05) from first half

Table 2 shows the positional differences for the metabolic power variables (P_met_, power zones, and HMLD) and distance variables (total distance, relative distance, high-speed distance, sprint distance, maximal speed, accelerations and decelerations). The P_met_ was lower in full backs and full forwards compared to half backs (ES = -1.03, -1.70 respectively), midfielders (ES = -1.22, -2.07 respectively) and half forwards (ES = -0.61, -1.28 respectively). The minimal power distance covered was *moderately* to *largely* greater in full backs compared to half backs (ES = 0.92), midfielders (ES = 1.47) and half forwards (ES = 0.99). Full forwards covered greater minimal power distance than half backs (ES = 0.67), midfielders (ES = 1.17) and half forwards (ES = 0.76). Positional differences also exist in low power to high power distance zones. Full backs covered a lower distance than half backs in intermediate power, moderate power and high power distance zones (ES = -1.30, -1.80, -1.73, respectively), than midfielders in low power, intermediate power, moderate power and high power zones (ES = -1.05, -1.49, -2.17, -1.86, respectively) and half forwards in low power, moderate power and high power zones (ES = -0.77, -0.88, -1.05, respectively) but greater distances than full forwards in intermediate power and moderate power (ES = 0.82, 0.71, respectively) zones. Half forwards covered lower distances in intermediate power, moderate power, and high power zones when compared to half backs (ES = -0.83, -1.14, -0.72 respectively) and midfielders (ES = -1.03, -1.48, -0.77 respectively). The half backs, midfielders and half forwards covered a greater high power distance compared to full backs (ES = 1.23, 1.37 and 0.84 respectively) and full forwards (ES = 1.77, 2.00 and 1.38 respectively). Similarly, these positions covered a greater relative high power distance than full backs (ES = 1.25, 1.25 and 0.75 for half backs, midfielders and half forwards respectively) and full forwards (ES = 1.75, 1.75 and 1.25 for half backs, midfielders and half forwards respectively). Half backs, midfielders and half forwards covered a greater HMLD than full backs (ES = -1.23, -1.37 and -0.84 respectively) and full forwards (ES = -1.77, -2.00 and -1.38 respectively).

**Table 2:** Metabolic power and distance variables with respect of position during elite hurling match-play. Data are presented as mean ± SD.

	Full Backs (*n* = 50)	Half Backs (*n* = 50)	Midfield (*n* = 50)	Half Forwards (*n* = 50)	Full Forwards (*n* = 50)
**Metabolic Power Variables**					
Average Metabolic Power (P_met_: W^·^Kg^-1^)	8.3 ± 1.7	9.9 ± 1.4 ^a^	10.0 ± 1.0 ^a^	9.2 ± 1.2 ^a^	7.6 ± 1.3 ^b^^c^^d^
MP Distance (m: > 0–5 W·kg^-1^)	1214 ± 224	1041 ± 145 ^a^	944 ± 132 ^a^	1016 ± 175^a^	1176 ± 248 ^b^^c^^d^
LP Distance (m: > 5–10 W·kg^-1^)	2155 ± 428	2351 ± 331	2597 ± 416 ^a^	2481 ± 423 ^a^	2228 ± 427 ^c^^d^
IP Distance (m: > 10–15 W·kg^-1^)	982 ± 234	1269 ± 207 ^a^	1317 ± 217 ^a^	1092 ± 219 ^b^^c^	818 ± 158 ^a^^b^^c^^d^
MDP Distance (m: > 15–25 W·kg^-1^)	1243 ± 349	1957 ± 439 ^a^	2027 ± 372 ^a^	1529 ± 299 ^a^^b^^c^	1021 ± 272 ^a^^b^^c^^d^
HP Distance (m: > 25 to 50 W·kg^-1^)	896 ± 234	1323 ± 259 ^a^	1321 ± 223 ^a^	1144 ± 237 ^a^^b^^c^	787 ± 212 ^b^^c^^d^
EP Distance (m: > 50 W·kg^-1^)	401 ± 123	362 ± 77	366 ± 92	405 ± 93	389 ± 84
HMLD (m: > 25 W·kg^-1^)	1301 ± 306	1680 ± 309 ^a^	1682 ± 249 ^a^	1545 ± 276 ^a^	1174 ± 260 ^b^^c^^d^
**Distance Variables**					
Total Distance (m)	6830 ± 1379	8399 ± 1043 ^a^	8566 ± 867 ^a^	7667 ± 1053 ^a^^b^^c^	6497 ± 1012 ^b^^c^^d^
Relative Distance (m·min^-1^)	98 ± 20	121 ± 14 ^a^	122 ± 12 ^a^	110 ± 15 ^a^^b^^c^	92 ± 15 ^b^^c^^d^
High-Speed Distance (m: > 17 km·h^-1^)	955 ± 201	1314 ± 241 ^a^	1348 ± 215 ^a^	1249 ± 189 ^a^	1048 ± 208 ^b^^c^^d^
Sprint Distance (m: > 22 km·h^-1^)	331 ± 98	320 ± 95	354 ± 76	368 ± 92	379 ± 88
Maximal Speed (km·h^-1^)	28.9 ± 2.7	28.8 ± 1.9	29.1 ± 1.6	29.4 ± 1.5	29.5 ± 2.5
Accelerations (> 2 m.s^-2^) (n)	128 ± 25	141 ± 26	121 ± 22 ^b^	132 ± 24	111 ± 17 ^a^^b^^d^
Decelerations (< 2 m.s^-2^) (n)	123 ± 22	142 ± 24 ^a^	120 ± 19 ^b^	119 ± 25 ^b^	97 ± 17 ^a^^b^^c^^d^

MP = Minimal Power; LP = Low Power; IP = Intermediate Power; MDP; Moderate Power; HP = High Power; EP = Elevated Power, HMLD = High Metabolic Load Distance; Diff = Difference.

^a^ Significantly different (p < 0.05) from full backs

^b^ Significantly different (p < 0.05) from half backs

^c^ Significantly different (p < 0.05) from midfielders

^d^ Significantly different (p < 0.05) from half forwards

Results comparing positions showed that full backs covered a *moderately* to *largely* lower total distance than half backs (ES = -1.28), midfielders (ES = -1.51) and half forwards (ES = -0.68). Half forwards covered a lower total distance than half backs (ES = -0.70) and midfielders (ES = -0.93) but greater total distance than full forwards (ES = 1.13). A lower total distance was covered by full forwards compared to half backs (ES = -1.85), midfielders (ES = -2.21) and half forwards (ES = -1.20). Half backs, midfielders and half forwards covered greater relative distances than full backs (ES = 1.33, 1.46 and 0.68, respectively) and full forwards (ES = 2.00, 2.21 and 1.20 respectively). Half forwards covered less relative distance than half backs (ES = -0.76) and midfielders (ES = -0.83). Half backs, midfielders and half forwards also outperformed full backs (ES = 1.62, 1.89 and 1.51, respectively) and full forwards (ES = 1.81, 1.42, 1.01, respectively) in high-speed distance. No positional differences were observed in total sprint distance and maximal speed. Half backs completed a greater number of accelerated efforts compared to midfielders (ES = 0.83). Full forwards performed a lower number of acceleration efforts compared to full backs (ES = -0.80), half backs (ES = -1.37) and half forwards (ES = -1.01). Half backs also had a *moderately* greater number of decelerations than full backs (ES = 0.83), midfielders (ES = 1.02), half forwards (ES = 0.94). Full forwards completed a lower number of decelerations than all other positions (ES = - 2.16, -1.03, respectively).

**Fig 2:**
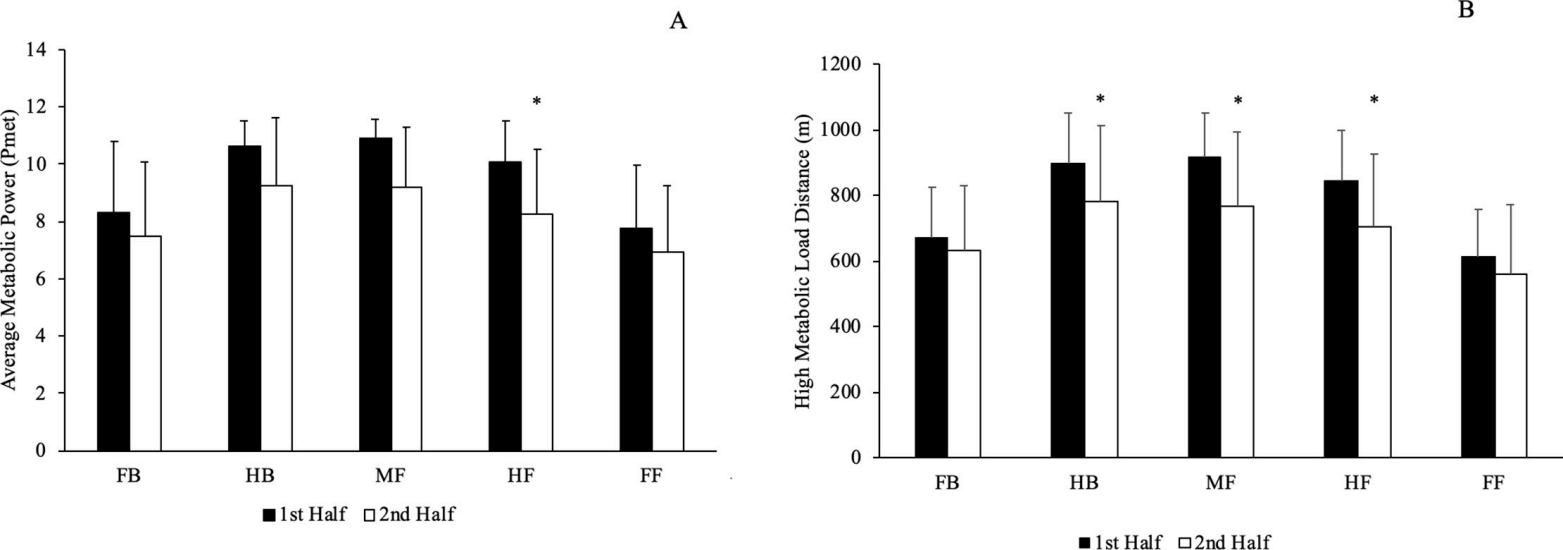
Mean ± SD temporal changes in average metabolic power and high metabolic load distance per position is shown. FB: full backs (N = 8), HB: half backs (N = 8), MF: midfielders (N = 5), HF: half forwards (N = 8) and FF: full forwards (N = 7). * Significant difference (p < 0.05) between halves.

Fig 2 depicts the temporal changes in P_met_ and HMLD by playing half. Half forwards experienced temporal decrements in P_met_ (ES = -0.33), EDI (ES = -0.50) in the second half. All other positions showed no temporal decrement in the second half for P_met_. Half backs (ES = -0.60), midfielders (ES = -0.81) and half forwards (ES = -0.74) covered a lower HMLD in the second half compared to the first half. Full backs and full forwards covered similar HMLD in both halves.

## Discussion

The metabolic characteristics of elite hurling match-play between positional groups and across halves of match-play are discovered for the first time. Therefore, to the best of the authors’ knowledge, the current investigation was the first study to provide estimates of the metabolic demands in hurlers during match-play. The main results showed that there were positional differences for all the metabolic power variables (P_met_, minimal power, low power, intermediate power, moderate power, high power distance, and HMLD) except for the distance covered in the elevated power distance zone. Furthermore, between-position differences were observed in total distance, relative distance, high-speed distance, accelerations and decelerations. There were second half decreases in all metabolic power metrics (P_met_, low power, intermediate power, moderate power, high power, elevated power, and HMLD) except minimal power distance and all GPS time-motion metrics (total distance, relative distance, high-speed distance, sprint distance, accelerations and decelerations) with the exception of max speed.

Previous studies in team sports have used the metabolic power variables to provide new insights into the physical demands of match-play [7,8,13,25]. The P_met_ ranged from 7.6 to 10.0 W·kg^−1^. The range is similar to those previously reported in Australian football [7] and Rugby League match-play [13] and soccer training [34] that used the same calculations as the current study. However, the range of P_met_ across positions in Gaelic football was slightly higher [8]. In Gaelic football, teams’ favor a more possession-based method of transferring the ball into attack and maintain possession until an opening appears in the defense so they can get close to the goal to kick a score [4]. In hurling, once the players hit the ball (< 70 m) they can slow down, whereas, in Gaelic football, players continue to run alongside the player in possession to receive a return pass.

The use of a metabolic power approach may help to indirectly quantify the energetic cost of changing speed in sport [7,8,13]. Full forwards performed a lower number of accelerations and decelerations than half backs, midfielders and half forwards, which may lead to a lower metabolic load being expended to change speed. Half backs, midfielders and half forwards had greater P_met_, HMLD, and distance covered in minimal power, intermediate power, moderate power and high power zones than full backs and full forwards. Since no previous data for metabolic power is known, a comparison with the hurling literature is not possible. Similar results were observed in Gaelic football where half backs, midfielders and half forwards performed greater high power activities [22]. The greater playing area and number of activities performed by half backs, midfielders and half forwards compared to full backs and full forwards may explain the differences between positions [35]. Indeed, the half backs, midfielders and half forwards role includes moving forward while in possession and backwards towards their own goals when opponents have possession. This may clarify why they cover greater distances compared to full backs and full forwards who stay close to the goal where the ball is hit towards them [1]. Half forwards covered lower intermediate power, moderate power and high power distance than half backs and midfielders. These differences may be attributed to the specific tactical role of the half forwards when play is restarted after a scoring attempt. During a match the goalkeeper strikes the ball back into the playing area (puck out) > 30 times [35], which is usually targeted towards the half forwards. A common tactical ploy used by half forwards is that once the puck out is about to be taken they start running to gain possession or create space for their teammates. The running action is usually of a constant speed. In contrast, quite often half backs and midfielders employ a zonal marking strategy where they may have to react as the ball enters their area and perform greater moderate power to high power efforts to gain possession before their opponent [2]. These specific tactical roles may have influenced the distance covered by each position.

Metabolic power variables across halves of play are presented here for the first time for hurling. All metabolic power variables decreased in the second half except minimal power distance [1–3]. As fatigue affected the distance covered above the low power threshold, the players could have slowed down and increased the distance covered at minimal power intensity in the second half. There were second half HMLD decrements in half backs, midfielders and half forwards while P_met_ temporal decrements only occurred in half forwards. These positions have been shown to cover greater HMLD compared to full backs and full forwards, which may have contributed to their specific drop-off between playing halves. The players in these positions may need to be substituted or switched in the full back or full forward position so they can cover less high demanding activities and minimize the individual or team performance drop-off.

Analyzing the GPS metrics, positional differences were observed, as reported in previous studies in hurling [1–5]. Specifically, full backs and full forwards covered a lower total distance, relative distance and high-speed distance than half backs, midfielders and half forwards, which is similar to previous studies in U17 [3] and U21 [2] hurling. The current results differed compared to a previous senior hurling study where full forwards covered the lowest total distance and relative distance compared to all other positions and full backs and full forwards covered lower high-speed distance than half backs, midfield and half forwards [1]. However, the previous study recruited only one team. Therefore, the lower running demands of full forwards may be due to this team’s specific tactical strategy. There was no difference in the total sprint distance between positions in the current study. These results are similar to previous research which examined the sprint demands of elite hurling [6]. In the present study the number of accelerations and decelerations for each position was lower than previously reported [4]. The difference in acceleration and deceleration zones thresholds may explain the difference in results. Current findings showed that half backs and full forwards performed the highest and lowest number of decelerations respectively when compared to all other positions. Half backs had a greater number of accelerations than midfielders and full forwards. In addition, full forwards performed a lower number of accelerations than full backs and half forwards. Half backs may have performed a greater number of accelerations due to their defensive role in running back towards their own goal to defend a goal scoring opportunity and rushing forward to deny a point scoring opportunity from long distance (< 80 m) [1]. Full forwards may have performed a lower number of accelerations due to the style of play implemented by the team, where they are located close to the goal and the ball is usually hit towards them.

The current analysis of metabolic power production provides useful additional information regarding the match-play demands of hurling. However, it is important to acknowledge the limitations associated with this approach. Firstly, although this paper focused on metabolic power metrics, the equivalent distance and estimated energy expenditure variables derived from GPS were not included here as these variables were shown to underestimate energy expenditure compared to a direct evaluation (metabolimeter) during exercise bouts and recovery phases [17]. Another limitation, which is common in studies that use GPS, is that match specific skills such as tackling were not accounted for. Therefore, the real energy cost of hurling cannot be estimated with accuracy without using direct measurements, which are not permitted during competitions. Thirdly, the direction of the locomotion activity (e.g. forwards, backwards or lateral) was not included in the present study as it has been shown to be unable to quantify such movements [36]. Therefore, further research is needed to evaluate the locomotor differences among positions. Future studies could utilise video tracking systems to add such information. Finally, this study provided mean data across the full duration of match-play. It has been shown that the ball is only in play for less than half a game [35]. Therefore, the ball-in-play match-play metabolic power demands may be higher than reported here. In addition, the traditional time-motion analysis has been shown to be far less when compared to the worst-case scenario running demands [5]. Future studies should assess the worst-case scenario metabolic power demands of hurling competition.

## Conclusions

The current study provides an insight into the metabolic power positional and between half demands of hurling match-play. Positional differences are shown in metabolic power variables with half backs, midfielders and half forwards appear to demonstrate increased activity profiles when compared to other positions. All metabolic power variables decreased in the second half except minimal power distance. Lastly, the present results suggest that the use of metabolic power to assess the running demands should be considered by coaches, especially during intermittent patterns of activities at low-speed running. Therefore, the integration of both metabolic power and GPS time-motion analysis metrics to describe the external load in hurling is recommended.

## Supporting information

S1 Table GPS output for one hurling game. The table shows sample GPS output for each player for one game.
(XLSX)
